# Hidden Treasure: Halophilic Fungi as a Repository of Bioactive Lead Compounds

**DOI:** 10.3390/jof10040290

**Published:** 2024-04-16

**Authors:** Shivankar Agrawal, Pruthviraj Chavan, Laurent Dufossé

**Affiliations:** 1 Indian Council of Medical Research (ICMR), V Ramalingaswami Bhawan, Ansari Nagar-AIIMS (All India Institute of Medical Sciences), Delhi 110029, India; 2 ICMR-National Institute of Traditional Medicine, Belagavi 590010, India; pruthvirajchavan716@gmail.com; 3 Chemistry and Biotechnology of Natural Products, ChemBioPro, Université de La Réunion, Ecole Supérieure d’Ingénieurs—Réunion, Océan Indien ESIROI Agroalimentaire, 97410 Saint-Denis, France

**Keywords:** halotolerant fungi, biomolecules, extreme, ecosystem, specialized metabolites

## Abstract

The pressing demand for novel compounds to address contemporary health challenges has prompted researchers to venture into uncharted territory, including extreme ecosystems, in search of new natural pharmaceuticals. Fungi capable of tolerating extreme conditions, known as extremophilic fungi, have garnered attention for their ability to produce unique secondary metabolites crucial for defense and communication, some of which exhibit promising clinical significance. Among these, halophilic fungi thriving in high-salinity environments have particularly piqued interest for their production of bioactive molecules. This review highlights the recent discoveries regarding novel compounds from halotolerant fungal strains isolated from various saline habitats. From diverse fungal species including *Aspergillus*, *Penicillium*, *Alternaria*, *Myrothecium*, and *Cladosporium*, a plethora of intriguing molecules have been elucidated, showcasing diverse chemical structures and bioactivity. These compounds exhibit cytotoxicity against cancer cell lines such as A549, HL60, and K-562, antimicrobial activity against pathogens like *Escherichia coli*, *Bacillus subtilis*, and *Candida albicans*, as well as radical-scavenging properties. Notable examples include variecolorins, sclerotides, alternarosides, and chrysogesides, among others. Additionally, several compounds display unique structural motifs, such as spiro-anthronopyranoid diketopiperazines and pentacyclic triterpenoids. The results emphasize the significant promise of halotolerant fungi in providing bioactive compounds for pharmaceutical, agricultural, and biotechnological uses. However, despite their potential, halophilic fungi are still largely unexplored as sources of valuable compounds.

## 1. Introduction

Environments with extreme conditions have typically been considered as malicious to be inhabited by organisms. The discovery of extremophiles changed this belief, and numerous micro-organisms, including the members of archaea, prokarya, and eukarya, have been reported in natural habitats with very high heat [1], cold, extreme pH, or high salinity. Those organisms exclusively growing in hypersaline conditions are called salt-loving organisms or halophiles. Hypersaline conditions are found in several locations across the globe, including littoral and arid regions as well as artificial salterns developed for mining salts [2]. Under hypersaline conditions, a water-limiting situation is generated due to the chemical binding of water to salt molecules. These water-limiting conditions prohibit the majority of life forms from growing in hypersaline environments; on the other hand, these are favorable conditions for halophiles to flourish. Most of the identified and studied extremophiles are archaea; however, other micro-organisms, such as bacteria, algae, and fungi, are also found in extreme environments. This wide variety of extremophilic organisms attracts the scientific community to investigate their uniqueness, which may enable inferring the evolutionary process of stress tolerance in other living forms. For the first time, the presence of eukaryotic fungi was reported in the hypersaline environment of the Dead Sea by [3] and in salterns by [4]. Since then, numerous novel species, including those once considered merely food contaminants, have been identified in hypersaline environments worldwide [5]. Researchers predominantly concentrate on bacteria, archaea, and algae when exploring extremophiles and extremozymes from saline environments, overlooking filamentous fungi, which remain largely unexplored [6]. However, extremophilic fungi hold significant promise in uncovering novel compounds given that fungi contribute over 40% of the active compounds derived from microorganisms [7,8,9,10,11,12,13,14]. This review aims to elucidate the ecological niche and adaptive strategies of halophilic fungi in natural hypersaline ecosystems, along with the pharmaceutical potential of their bioactive metabolites for prospective drug development.

## 2. Physiology of Halophilicity in Fungi

Fungi living under hypersaline conditions possess a specialized physiology that enables them to flourish in such conditions that are considered harsh for other organisms. The hypersaline environment imposes two major limitations inhibiting the growth of organisms: (1) high osmolarity and (2) ionic toxicity due to the high ionic concentration. The hypersaline conditions lead to high osmolarity, resulting in very low water activity. This low water activity is generated due to the bonding of water molecules (H_2_O) with salt (NaCl). Thus, water activity is one of the most determinative factors for the growth of fungal life under hypersaline conditions. The mitigation strategies of halophiles to survive and grow in hypersaline environments mainly include maintaining a low water potential condition in comparison to their surrounding environment. These mitigation strategies involve several physiological mechanisms, such as selective modification of the fluidity of the plasma membrane, generation and accumulation of compatible solutes, and the high osmolarity glycerol (HOG) pathway [15,16,17].

## 3. Ecology and Biodiversity of Halophilic Fungi

Similar to other abiotic factors such as temperature, water, and light, salinity also imposes deleterious effects on the growth and development of organisms. The water activity of saline environments defines the extent of adverse impacts on organisms; however, some fungi belonging to different phylogenetic origins growing in hypersaline conditions showed their halotolerant and halophilic nature [18]. Research indicates that halophilic fungi thrive in various hypersaline environments worldwide, including regions like Slovenia, Romania, Thailand, China, India, Brazil, and others [19,20,21,22]. These fungi have been found in habitats with diverse salinity levels, such as marine environments (sea water, marine plants, and mangroves) [23], solar salterns used for salt production through seawater evaporation [24], natural salt lakes, salt mines [25,26], saline soil, salt deserts, sebkhas (resulting from salt lake evaporation, characterized by a range of soluble salts) [27], and high-salt-content foods and fermented products [28]. Kirk et al. [18] noted that, among the 106 existing orders of fungi, 10 were identified as tolerant to low water activity (a_w_). Halophilic or halotolerant characteristics were observed in certain orders, including *Wallemiales*, *Trichnosporales*, and *Sporidiales* within Basidiomycota, and *Capnodiales*, *Eurotiales*, and *Dothideales* within Ascomycota. Various yeast species such as *Rhodotorula*, *Debaryomyces*, *Aureobasidium*, and *Trichosporum*, along with filamentous fungi like *Cladosporium*, *Scopulariopsis*, *Alternaria*, and species of *Aspergillus* and *Penicillium*, were described as halotolerant. Halophilic species were identified within genera such as *Wallenia*, *Hortea*, *Phaetotheca*, and *Trimmatostroma* [29,30]. Several studies have consistently reported the presence of numerous fungi in hypersaline water and environments across different continents [4]. Fungi from orders including Dothideales, Capnodiales, and Eurotiales of Ascomycota, as well as Wallemiales and Tremellales of Basidiomycota, demonstrated halotolerance, with black yeasts being particularly prominent in the hypersaline water of salterns [31]. These yeasts have melanized cell walls, enabling them to withstand high-salt-stress conditions [32]. Notably, among black yeasts, *Hortaea werneckii* is widely distributed across saline environments, thriving optimally at 3.0–4.5 M NaCl [5]. *Wallemia ichthyophaga* (Basidiomycetes) stands out as a genuinely halophilic fungus requiring a minimum of 10% NaCl for growth [33,34].

## 4. Role in Production of Therapeutic Compounds

**Figure 1:**
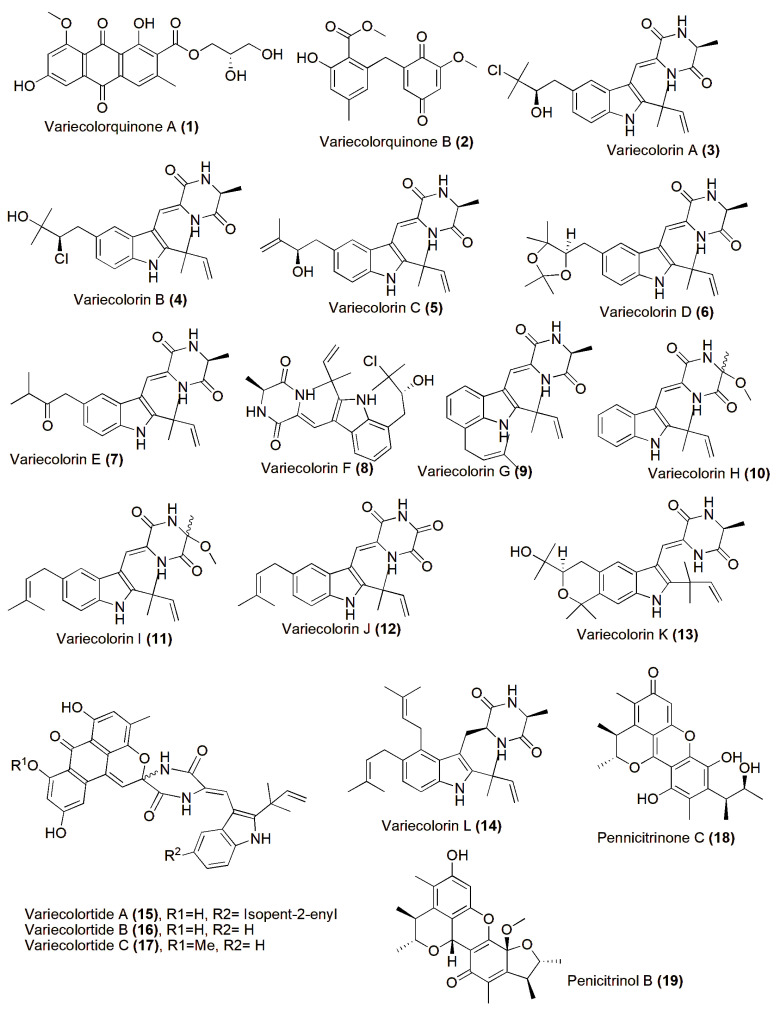
Chemical structures of biologically active and novel compounds isolated from halophilic/halotolerant fungi (**1**–**19**).

Halophiles, organisms capable of surviving in high-salinity conditions and sometimes requiring salt for growth, span all domains of life [35], predominantly represented by bacteria, archaea, algae, and fungi [36]. These microorganisms produce various intriguing biomolecules with distinctive properties suitable for diverse industrial applications. Halophilic fungi, in particular, continue to be unearthed, yielding novel compounds regularly. A variety of novel compounds have been discovered from the halotolerant fungal strain *Aspergillus variecolor* B-17, which was isolated from sediments collected in the Jilantai salt field, Alashan, Inner Mongolia, China. Among these compounds are Variecolorquinones A–B (**1**–**2**), two new quinone-type compounds showing cytotoxic activity against A549 cells (compound **1**, IC_50_ 3.0 µM), HL60 cells (compound **2**, IC_50_ 1.3 µM), and P388 cells (compound **2**, IC_50_ 3.7 µM) [37]. Variecolorins A–L (**3**–**14**) showed no cytotoxicity against the P388, HL-60, BEL-7402, and A-549 cell lines. However, compounds A–K (**3**–**13**) exhibited weak radical-scavenging activity against DPPH, with IC_50_ values ranging from 75 to 102 µM [38]. Variecolortides A–C (**15**–**17**) shared a unique ‘spiro-anthronopyranoid diketopiperazine’ structure with a stable hemiaminal function. All three compounds showed weak cytotoxic activity against the K-562 cell line, with IC_50_ values of 61, 69, and 71 µM, respectively (the positive control paclitaxel IC_50_ 0.93 µM), and slight radical-scavenging activity against the DPPH radical, with IC_50_ values of 63, 84, and 91 µM, respectively (the positive control vitamin C IC_50_ 22 µM) [39]. Pennicitrinone C (**18**) and penicitrinol B (**19**), two new citrinin dimers, were produced by the halotolerant fungal strain *Penicillium citrinum* B-57, obtained from sediments in the Jilantai salt field, Alashan, Inner Mongolia, China. Compound **18** scavenged DPPH radicals with an IC_50_ value of 55.3 µM (the positive control L-ascorbic acid IC_50_ 22.7 µM) but exhibited no cytotoxic activity against the P388, A549, BEL7402, or HL60 cell lines (IC_50_ > 50 µM) [40] (Figure 1).

**Figure 2:**
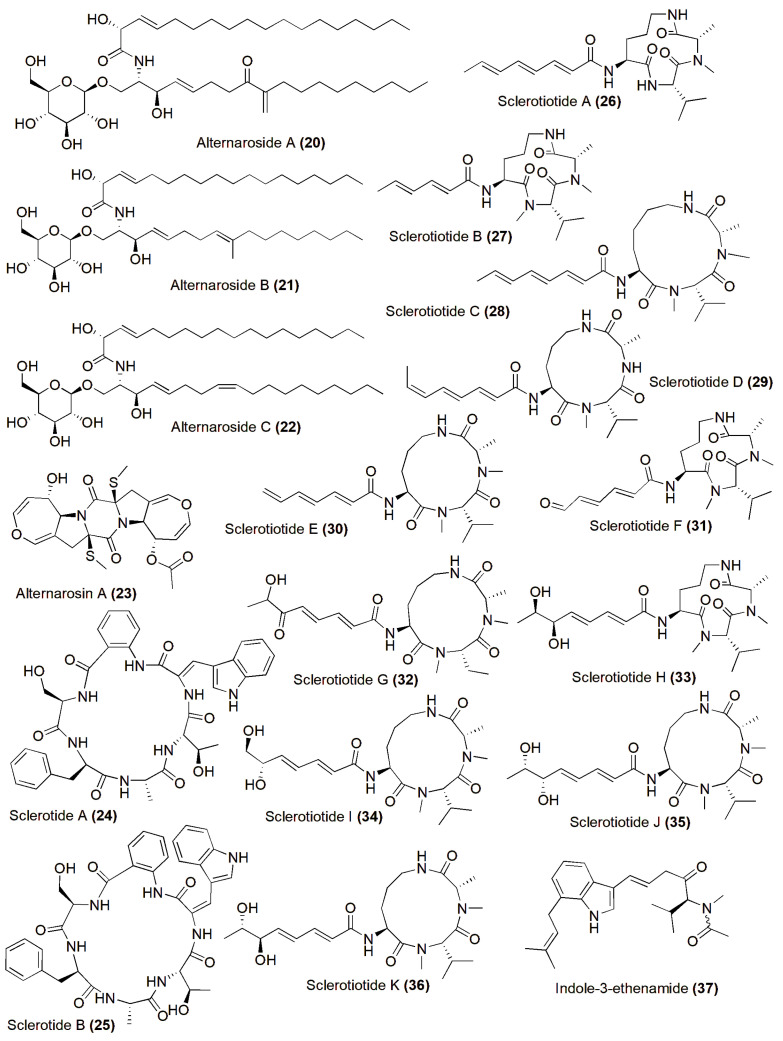
Chemical structures of biologically active and novel compounds isolated from halophilic/halotolerant fungi (**20**–**37**).

The halotolerant fungus *Alternaria raphanin* THW-18, isolated from sediment in the Hongdao Sea salt field, China, yielded three new cerebrosides, alternarosides A–C (**20**–**22**), and a novel diketopiperazine alkaloid, alternarosin A (**23**). These compounds exhibited antimicrobial activity against *Escherichia coli*, *Bacillus subtilis*, and *Candida albicans*, with MIC values ranging from 70 to 400 µM. However, no cytotoxicity against the P388, HL-60, A549, and BEL-7402 cell lines or DPPH radical-scavenging activity were observed [41]. The halotolerant *Aspergillus sclerotiorum* PT06-1, isolated from the Putian Sea salt field, China, produced novel cyclic hexapeptides, sclerotides A–B (**24**–**25**), in a nutrient-limited hypersaline medium. These compounds showed inhibition against *C. albicans*, with MIC values of 7.0 and 3.5 µM, respectively. Compound **25** also displayed weak cytotoxic activity against HL-60 cells (IC_50_ 56.1 µM) and antibacterial activity against *Pseudomonas aeruginosa* (MIC 35.3 µM) [42]. Furthermore, eleven new aspochracin-type cyclic tripeptides named sclerotiotides A–K (**26**–**36**) [43] and one new cytotoxic indole-3-ethenamide (**37**) from the same halotolerant fungal strain were isolated in a nutrient-rich hypersaline medium. Among these compounds, **26**, **27**, **31**, and **34** exhibited antifungal activity against *C. albicans*, with MIC values ranging from 3.8 to 30 µM. Compound **37** demonstrated cytotoxic activity against A-549 and HL-60 cells, with IC_50_ values of 3.0 and 27 µM, respectively. Additionally, compounds **35**–**36** were identified as isomers with the same molecular formula, and NMR data suggested enantiotopic differences in the fatty acid moiety for 33/35 and 34/36, respectively [44] (Figure 2).

**Figure 3:**
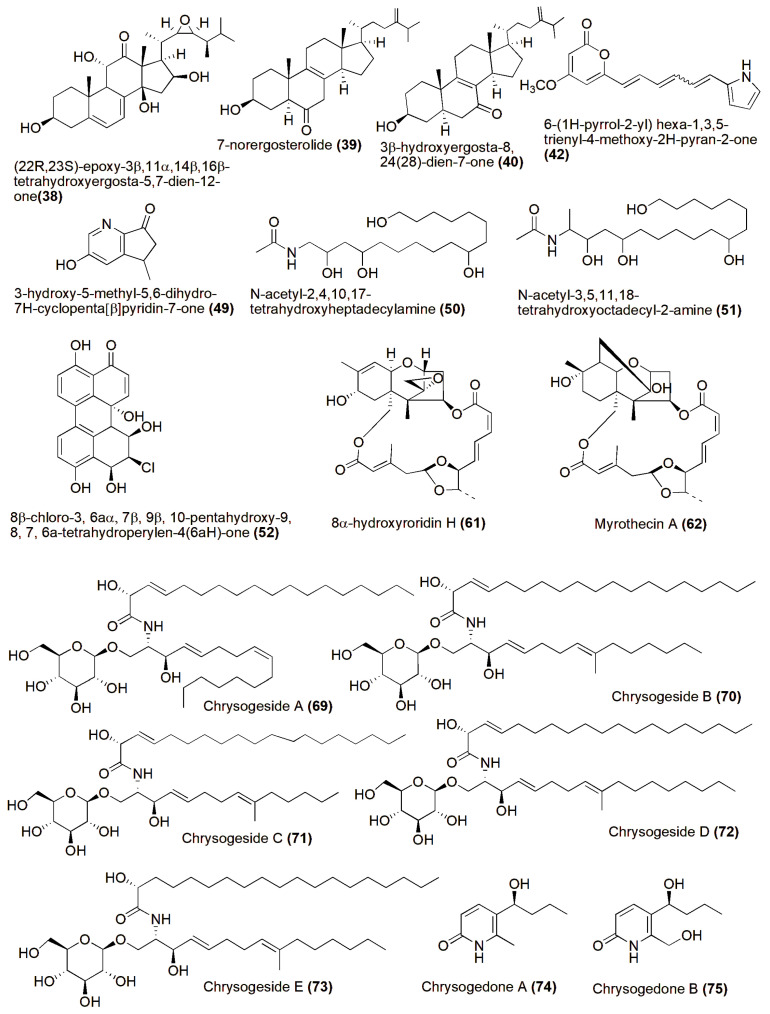
Chemical structures of biologically active and novel compounds isolated from halophilic/halotolerant fungi (**38**–**75**).

Examination of the EtOAc extract from the fermentation broth of the halotolerant fungus *Aspergillus flocculosus* PT05-1 under 10% salinity led to the discovery and characterization of several compounds. Among these were a novel derivative of ergosterol (**38**), a new red pyrrole pigment (**42**), and nine previously identified compounds: 7-norergosterolide (**39**), 3β-hydroxyergosta-8, 24(28)-dien-7-one (**40**), cerevisterol (**41**), (2R,3E)-2-hydroxy-N-[(2S,3R,4E,8E)-l-β-D-glucopyranosyloxy-3-hydroxy-9-methylnonadec-4,8-dien-2-yl] heptadec-3-enamide (**43**), (2R)-2-hydroxy-N-[(2S,3R,4E,8E)-l-β-D-glucopyranosyloxy-3-hydroxy-9-methylnonadec-4,8-dien-2-yl]heptadecanamide (**44**), cerebroside C (**45**), 4-(1H-pyrrol-2-yl)-1-isoquinolone-3-carboxylic acid (**46**), neoaspergillic acid (**47**), and hydroxyneoaspergillic acid (**48**). The new compound **38**, along with 7-norergosterolide (**39**) and 3β-hydroxyergosta-8,24(28)-dien-7-one (**40**), exhibited cytotoxic effects against HL-60 and BEL-7402 cells, with IC_50_ values ranging from 12 to 18 μM, and antimicrobial activity against *Enterobacter aerogenes*, *P. aeruginosa*, and *C. albicans*, with MIC values ranging from 1.6 to 15 μM, respectively. Compound **42** demonstrated antibacterial properties against *E. aerogenes*, with an MIC value of 3.7 μM [45]. A novel cyclopentanopyridine alkaloid, named 3-hydroxy-5-methyl-5,6-dihydro-7H-cyclopenta[β]pyridin-7-one (**49**), was discovered alongside 11 previously identified aromatic compounds in the secondary metabolites of the halotolerant fungal strain *Wallemia sebi* PXP-89, cultivated in a 10% NaCl medium. Compound **49** demonstrated antimicrobial efficacy against *Enterobacter aerogenes*, with a minimum inhibitory concentration (MIC) of 76.7 μM [46]. Two novel amides, denoted as N-acetyl-2,4,10,17-tetrahydroxyheptadecylamine (**50**) and N-acetyl-3,5,11,18-tetrahydroxyoctadecyl-2-amine (**51**), were discovered from a halotolerant fungus, *Myrothecium* sp. GS-17. They were isolated using the dilution isolation method from soil samples collected from a saline area in Gansu Province. The cytotoxicity of these compounds against cancer cells was assessed, revealing that compound **51** exhibited mild activity against human leukemia (HL-60) cells, with an IC_50_ value of 63.61 μM [47]. A new perylenequinone (**52**), 8β-chloro-3, 6aα, 7β, 9β, 10-pentahydroxy-9, 8, 7, 6a-tetrahydroperylen-4(6aH)-one, along with eight known compounds, alterperylenol (**53**), dihydroalterperylenol (**54**), adenine (**55**), adenosine (**56**), deoxyadenosine (**57**), guanosine (**58**), tryptophan (**59**), and hexadecanoic acid (**60**), was isolated and identified from a halotolerant fungus, *Alternaria* sp. M6 [48]. Two novel trichothecenes, designated as 8α-hydroxyroridin H (**61**) and myrothecin A (**62**), were discovered alongside six previously identified compounds, namely 8α-acetoxy roridin H (**63**), isororidin K (**64**), verrucarin A (**65**), verrucarin J (**66**), verrucarin L (**67**), and 8α-acetoxy verrucarin L (**68**). These compounds were extracted from the fermentation broth of a halotolerant fungus identified as *Myrothecium* sp. GS-17, which was isolated from a soil sample obtained from a saline environment. Notably, compounds **61** and **62** exhibited activity against plant pathogenic fungi, specifically *Rhizoctonia solani* and *Fusarium oxysporum* [49]. Chrysogesides A–E (**69**–**73**), five new cerebrosides, and chrysogedones A and B (**74**–**75**), two novel 2-pyridone alkaloids, were discovered in the fermentation broth of *Penicillium chrysogenum* PXP-55. This halotolerant fungus was cultivated in a hypersaline medium. Notably, chrysogesides B–D represent the first cerebrosides containing an unsaturated C19 fatty acid. Compound **70** exhibited antimicrobial properties against *Enterobacter aerogenes*, displaying a minimum inhibitory concentration (MIC) of 1.72 μM [50] (Figure 3).

**Figure 4:**
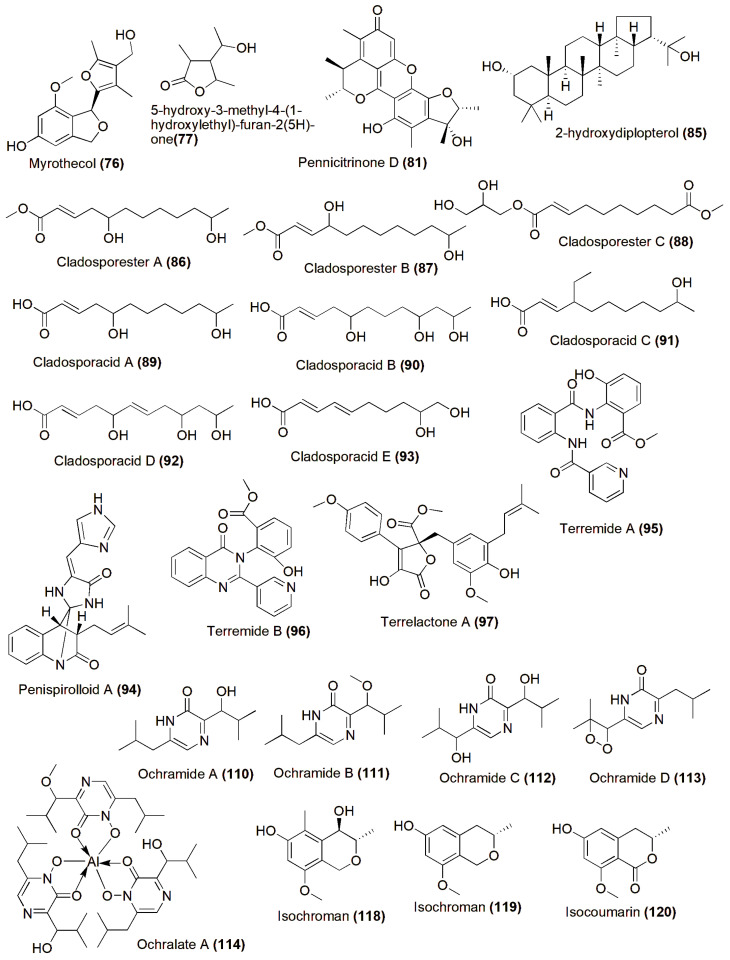
Chemical structures of biologically active and novel compounds isolated from halophilic/halotolerant fungi (**76**–**120**).

A halotolerant fungus, *Myrothecium* sp. GS-17, was the source of two new polyketides, myrothecol (**76**) and 5-hydroxy-3-methyl-4-(1-hydroxylethyl)-furan-2(5H)-one (**77**), along with three known compounds, 5-hydroxyl-3-[(1S)-1-hydroxyethyl]-4-methylfuran-2(5H)-one (**78**), 3,5-dimethyl-4-hydroxylmethyl-5-methoxyfuran-2(5H)-one (**79**), and 3,5-dimethyl-4-hydroxymethyl-5-hydroxyfuran-2(5H)-one (**80**). Compound **76** represents the first naturally occurring polyketide featuring a distinctive furylisobenzofuran skeleton [51]. A strain of halotolerant fungi identified as *Penicillium notatum* B-52 exhibited the production of cytotoxic metabolites against the mouse temperature-sensitive cdc2 mutant cell line tsFT210. This strain was isolated from salt sediments gathered in Qinghai Lake, Qinghai, China. Through bioassay-guided fractionation, researchers were able to isolate and identify a novel citrinin dimer, named pennicitrinone D (**81**), alongside three known compounds: pennicitrinone A (**82**), citrinin (**83**), and mycophenolic acid (**84**) [52]. A novel pentacyclic triterpenoid known as 2-hydroxydiplopterol (**85**) was discovered within the metabolites generated by the halotolerant fungal strain *Aspergillus variecolor* B-17. Furthermore, 2-hydroxydiplopterol demonstrated cytotoxic effects against K562 cells, displaying an IC_50_ value of 22 µM [53]. *Cladosporium cladosporioides* OUCMDZ-187, a fungus tolerant to high salt levels (halotolerant), was discovered in the mangrove plant *Rhizophora stylosa* obtained from Shankou, Guangxi Province, China. From the fermentation broth of OUCMDZ-187 cultivated in a hypersaline medium (10% salt), three novel fatty acid esters named cladosporesters A–C (**86**–**88**) and five new fatty acids labeled cladosporacids A–E (**89**–**93**) were isolated in the ethyl acetate extract. However, none of these compounds exhibited cytotoxic effects against three different cancer cell lines (IC_50_ > 50 μM) or demonstrated any antimicrobial activity (MIC > 150 μM) [54]. Penispirolloid A (**94**) is a newly discovered alkaloid with a distinctive spiro-imidazolidinyl skeleton, extracted from a halotolerant fungal strain identified as *Penicillium* sp. OUCMDZ-776. This compound exhibited notable antifouling properties against *Bugula neritina* larvae, demonstrating an EC_50_ value of 2.40 μg/mL [55]. *Aspergillus terreus* PT06-2, a fungus isolated from the sediment of the Putian Sea Saltern in Fujian, China, yielded three novel compounds: terremides A (**95**) and B (**96**), and terrelactone A (**97**), alongside twelve known compounds (**98**–**109**). The newly discovered compounds **95** and **96** demonstrated antibacterial properties against *Pseudomonas aeruginosa* and *Enterobacter aerogenes*, with minimum inhibitory concentration (MIC) values of 63.9 and 33.5 μM, respectively. Compound **99** exhibited moderate activity against H1N1 and showed reduced cytotoxicity, with IC_50_ and CC_50_ values of 143.1 and 976.4 μM, respectively [56]. Five novel pyrazin-2(1H)-one derivatives, named ochramides A–D (**110**–**113**) and ochralate A (**114**), along with three previously identified analogues (**115**–**117**), were extracted from the culture broth of halotolerant *Aspergillus ochraceus* LCJ11-102, obtained from marine coral, cultivated in a nutrient-restricted medium containing 10% NaCl. Compounds **111**, **114**, and **115** exhibited antimicrobial activity against *Enterobacter aerogenes*, with minimum inhibitory concentration (MIC) values of 40.0 µM, 18.9 µM, and 20.1 µM, respectively [57]. The ethyl acetate extract of *Penicillium* sp., isolated from the sediment of Egypt’s hypersaline lake Wadi El-Natrun, underwent chromatographic analysis, yielding two new isochromans (**118**–**119**), a novel isocoumarin derivative (**120**), and six known compounds (**121**–**126**). Despite screening against *Staphylococcus aureus* ATCC 29213, *Streptococcus pneumonia* ATCC 49619, and *Escherichia coli* ATCC 25922 at a concentration of 64 mg/mL, none of the isolated compounds demonstrated significant antimicrobial activity. Furthermore, when tested against the murine lymphoma L5178Y cell line, all the compounds exhibited mild to moderate cytotoxic effects [58] (Figure 4).

## 5. Conclusions and Perspective

Within the captivating domain of extreme environments, where salinity reigns supreme, a fascinating assembly of organisms arise as resilient conquerors—halotolerant and halophilic fungi. Residing in saline habitats, these fungi defy the odds, thriving in conditions that test the fundamental essence of life. It has been theorized that such extreme environments, characterized by high salt concentrations, could awaken dormant genes and activate unique biosynthesis pathways, potentially leading to the production of structurally distinctive and biologically active secondary metabolites. Consequently, these environments serve as fertile grounds for the discovery of novel compounds and enzymes. The ability of extremophiles to conduct biochemical reactions under extreme conditions offers them a distinct advantage in the production of fuels and chemicals.

Recent advancements in biotechnology have significantly enhanced our understanding of halophilic fungi and their potential applications through the development of innovative methods for characterizing bioactive compounds. Techniques such as metabolomics, genomics, and bioinformatics have enabled researchers to delve deeper into the intricate metabolic pathways of these fungi, uncovering novel bioactive compounds with promising implications across various fields, including biotechnology, agriculture, and medicine. By deciphering the genomes of these organisms, researchers can lay the groundwork for uncovering new biochemical processes, developing innovative applications and products, and gaining insights into the mechanisms by which organisms overcome abiotic stresses. By leveraging advanced analytical tools and multiomics, scientists have overcome challenges associated with studying halophilic fungi, such as difficulties in cultivation and metabolite extraction. Researchers have found that the multiomics approach provides a cost-effective, comprehensive, structured, and interactive overview of biological mechanisms to explore the spectrum from key transcriptional players in the regulation of secondary metabolite biosynthesis and its epigenetic control to approaches for the detection of new gene clusters and substances by genome mining, metagenomics/metatranscriptomics, and metabolomics to the use of secondary metabolite profiles in fungal chemotaxonomy [59].

For instance, Zhou et al. utilized transcriptomics to investigate the expression of the genes involved in the bioactive compound biosynthesis in the medicinal fungi Sanghuang. Their study demonstrated how the multiomics approach offers a cost-effective and comprehensive understanding of biological mechanisms, ranging from transcriptional regulation to genome mining and metabolomics analysis for the detection of new gene clusters and substances [60]. Similarly, Gonçalves et al. employed untargeted metabolomics coupled with genome sequencing to explore the chemical diversity of *Emericellopsis cladophorae* MUM 19.33, revealing a rich repertoire of genes encoding various enzymes, transporters, and secondary metabolite biosynthetic gene clusters. This integrated approach shed light on the resilience mechanisms of fungi against harsh environmental conditions, including the biosynthesis of osmolytes and ion transport systems [61]. Additionally, Gómez et al. pioneered the use of multiomics, specifically transcriptomics and metabolomics, to compare the saturation and optimal concentrations of salt for halophilic *Aspergillus sydowii* fungi. This interdisciplinary collaboration provided valuable insights into the adaptation mechanisms of halophilic fungi to saline environments [62]. Overall, the integration of multiomics and interdisciplinary collaboration is crucial for fully exploring the potential of halophilic fungi for bioactive metabolites. These innovative approaches not only facilitate the identification and isolation of bioactive compounds but also contribute to a deeper understanding of the ecological functions and survival strategies of halophilic fungi.

This review delves deeply into the intricacies of salt-loving fungi, probing their unique adaptations and revealing the diverse array of secondary metabolites that hold the potential to revolutionize our understanding of biology and medicine. For instance, the halotolerant fungal strain *Aspergillus variecolor* B-17, sourced from sediments in the Jilantai salt field, Inner Mongolia, China, has unveiled several intriguing compounds. Variecolorquinones A–B exhibited cytotoxic activity against cancer cell lines, while Variecolorins A–L displayed radical-scavenging properties. Similarly, Variecolortides A–C demonstrated cytotoxic and radical-scavenging activity. Pennicitrinone C and penicitrinol B showcased radical-scavenging abilities. The halotolerant fungus *Alternaria raphanin* THW-18, isolated from a sea salt field in China, produced cerebrosides and a diketopiperazine alkaloid with antimicrobial activity. *Aspergillus sclerotiorum* PT06-1 yielded cyclic hexapeptides and cyclic tripeptides with antifungal and cytotoxic properties. Moreover, the halotolerant fungal strain *Aspergillus flocculosus* PT05-1 generated compounds including a derivative of ergosterol and a red pyrrole pigment with cytotoxic and antimicrobial effects. Similarly, *Wallemia sebi* PXP-89 produced a cyclopentapyridine alkaloid with antimicrobial efficacy. Compounds such as N-acetyl-2,4,10,17-tetrahydroxyheptadecylamine and N-acetyl-3,5,11,18-tetrahydroxyoctadecyl-2-amine exhibited cytotoxicity against cancer cells, while others like perylenequinone showed promise against plant pathogenic fungi. Halotolerant fungi such as *Penicillium chrysogenum* PXP-55 and *Myrothecium* sp. GS-17 unveiled cerebrosides, alkaloids, and polyketides with antimicrobial activity. A variety of novel compounds with antimicrobial and cytotoxic properties were also discovered from *Penicillium notatum* B-52, *Aspergillus terreus* PT06-2, *Aspergillus ochraceus* LCJ11-102, and *Penicillium* sp. from Wadi El-Natrun, Egypt.

In summary, these findings underscore the vast industrial potential of halotolerant fungi, offering a rich source of bioactive compounds for pharmaceutical, agricultural, and other industrial applications. Further exploration and development of these compounds could lead to valuable products with diverse commercial uses. Despite the myriad of advantages and vast potential they offer, the mycobiota of saline environments remain largely unexplored, suggesting that numerous biomolecules with exceptional properties may still lie concealed within. Hence, further research on halophilic fungi is imperative to fully harness their potential.

## Data Availability

Not applicable.
